# Milieu for Endothelial Differentiation of Human Adipose-Derived Stem Cells

**DOI:** 10.3390/bioengineering5040082

**Published:** 2018-10-03

**Authors:** Kendra Clark, Amol V. Janorkar

**Affiliations:** Department of Biomedical Materials Science, School of Dentistry, University of Mississippi Medical Center, Jackson, MS 39216, USA; kreed2@umc.edu

**Keywords:** Human adipose-derived stem cells, hydrogel, endothelial cells, scaffold-free culture

## Abstract

Human adipose-derived stem cells (hASCs) have been shown to differentiate down many lineages including endothelial lineage. We hypothesized that hASCs would more efficiently differentiate toward the endothelial lineage when formed as three-dimensional (3D) spheroids and with the addition of vascular endothelial growth factor (VEGF). Three conditions were tested: uncoated tissue culture polystyrene (TCPS) surfaces that induced a 2D monolayer formation; elastin-like polypeptide (ELP)-collagen composite hydrogel scaffolds that induced encapsulated 3D spheroid culture; and ELP-polyethyleneimine-coated TCPS surfaces that induced 3D spheroid formation in scaffold-free condition. Cells were exposed to endothelial differentiation medium containing no additional VEGF or 20 and 50 ng/mL of VEGF for 7 days and assayed for viability and endothelial differentiation markers. While endothelial differentiation media supported endothelial differentiation of hASCs, our 3D spheroid cultures augmented this differentiation and produced more von Willebrand factor than 2D cultures. Likewise, 3D cultures were able to uptake LDL, whereas the 2D cultures were not. Higher concentrations of VEGF further enhanced differentiation. Establishing angiogenesis is a key factor in regenerative medicine. Future studies aim to elucidate how to produce physiological changes such as neoangiogenesis and sprouting of vessels which may enhance the survival of regenerated tissues.

## 1. Introduction

Dental infection, disease, or aging can lead to complex soft and hard tissue defects. The oral cavity does not readily regenerate the loss of soft and hard tissue, neither does it return to its original form. Instead, the lost tissue is replaced by a fibrous network with no restoration in form or function. In order to restore form and function, regeneration must take place by grafting the tissue [1]. Autogenous bone is the gold standard for regeneration of hard tissue defects. Autogenous bone has the critical properties for regeneration such as osteoinductivity, osteogenicity, and osteoconductivity. The disadvantages of an autogenous bone graft are a second surgical site and resorption over time. Alternative strategies range from alloplastic bone grafting to the application of growth factors to stem cells supported by biodegradable scaffolds for creating elaborate three-dimensional (3D) constructs for tissue regeneration.

Various approaches have been considered in tissue engineering and regenerative medicine, but currently, the most common is to use a biodegradable scaffold in the shape of the new tissue that is seeded with either stem cells or autologous cells from biopsies of damaged tissues. Several biomaterials can be chosen when designing a scaffold, including naturally occurring macromolecules such as collagen, alginate, agarose, hyaluronic acid derivatives, chitosan, and fibrin, and man-made polymers such as polyglycolic acid (PGA), polylactic acid (PLA), poly(caprolactone), poly(dioxanone), and poly(glycerol-sebacate) [2]. Recently, hydrogel scaffolds have received considerable attention due to their unique compositional and structural similarities to the natural extracellular matrix (ECM) in addition to their desirable framework for cellular proliferation and survival [3]. Hydrogels are 3D networks composed of hydrophilic polymers cross-linked either through covalent bonds or held together via physical intramolecular and intermolecular attractions. However, precise control over hydrogel properties, such as porosity, pore size, and pore interconnectivity, remains a challenge [4]. Hydrogels scaffolds may be prepared in various ways. These include one-step procedures like polymerization and parallel cross-linking of multifunctional monomers, as well as multistep procedures involving synthesis of polymer molecules with reactive groups and their subsequent cross-linking. Collagen hydrogels have been used successfully as 3D substrates for cell culture and have shown promise as scaffolds for engineered tissues. Collagen does not induce toxicity-based inflammatory or immunological responses when grafted because it is biocompatible, biodegradable, and already present in the body [5,6,7,8].

While scaffolds can support cells encapsulated in a 3D microenvironment, they often restrict the cell proliferation and differentiation due to mechanical restriction they place on growing cells. Therefore, scaffold-free cultures that also achieve 3D culture conditions are gaining popularity. In particular, 3D spheroid cultures have been shown to enable enhanced differentiation and long-term culture in multiple cell types, including liver, adipose, and bone cells. For instance, by culturing primary hepatic cells in 3D spheroid conditions, these cells were more differentiated and maintained viability during long-term cultures of at least 5 weeks [9,10]. 3D spheroid culture system contributes to an optimization for efficient differentiation of mesenchymal stem cells (MSCs) to adipocytes and osteoblasts [11,12]. Furthermore, osteogenic differentiation of MSCs exhibits enhanced bone regeneration in calvarial defects in rats in comparison to monolayer culture [13].

The extracellular matrix proteins elastin and collagen play an essential role in rendering elasticity and mechanical strength to blood vessels [14]. However, insufficient vascularization is a persistent issue in 3D culture for tissue engineering. Vascularization is crucial for tissue regeneration. Endothelial cells and their paracrine factors, like vascular endothelial growth factor (VEGF), mediate the formation of vasculature into engineered tissues. Endothelial cells are a vital component of the capillaries that provide blood supply and excretion of waste in tissues. We hypothesized that human adipose-derived stem cells (hASCs) would differentiate toward the endothelial lineage with the addition of endothelial differentiation medium. Furthermore, we hypothesized that the hASCs would more efficiently do so when formed as 3D spheroids. Therefore, the aims of this study were (1) to examine the ability of the hASCs to differentiate into endothelial cells in our 3D spheroid culture systems and (2) to determine if the addition of VEGF can further enhance endothelial cell differentiation.

## 2. Materials and Methods

### 2.1. Expression, Purification, and Chemical Modification of Elastin-Like Polypeptides (ELPs)

ELPs with a primary sequence (Valine-Proline-Glycine-Valine-Glycine)_40_, referred herein as ELP1, and (Valine-Proline-Glycine-Valine-Glycine)_120_, referred herein as ELP2, were produced as previously described [15,16]. The ELP1 (molecular weight = 17,000 Da) was chemically conjugated to branched polyethyleneimine (PEI, molecular weight = 800 Da; Sigma, St. Louis, MO, USA) using carbodiimide chemistry as previously described [15]. The ELP2 (molecular weight = 51,000 Da) was used to form hydrogels as described elsewhere [16,17].

### 2.2. ELP1-PEI Coating

ELP1-PEI coating masks the underlying adherent tissue culture polystyrene (TCPS, Corning, Lowell, MA) and alters cell morphology and differentiation. Five mol% ELP1-PEI conjugate was adsorbed to 24-well TCPS plates using deionized water as solvent, previously identified as optimal for spheroid formation in 3T3-L1 mouse preadipocytes [18] and successful in forming hASC spheroids [19,20]. Plates were incubated at 37 °C for 48 h to remove all deionized water solvent.

### 2.3. Hydrogel Formation

The preparation of collagen and ELP2-collagen composite hydrogels has previously been described [17]. In brief, hydrogels were prepared by varying concentrations of collagen type I (rat tail, Invitrogen, Carlsbad, CA, USA) at 2 and 6 mg/mL (referred to as 2C and 6C hydrogels), adding ELP2 to 2 mg/mL collagen at 3:1 mass ratio (referred to as 2C+E hydrogel), or cross-linking the hydrogels utilizing ethyl (dimethylaminopropyl) carbodiimide (EDC) and *N*-Hydroxysuccinimide (NHS) (referred to as 2C_C, 6C_C, and 2C+E_C hydrogels). The final solution was incubated in a 96-well TCPS plate at 37 °C and >70% humidity for 18 h to form a hydrogel.

### 2.4. Cell Culture

Stromal derived vascular fractions were isolated from elective liposuction aspirates under an IRB-approved protocol (# 2012-0004) at the University of Mississippi Medical Center. Cells from the isolation were confirmed to be CD34+ using flow cytometry and are then referred to as hASCs. Forty-thousand cells were seeded per hydrogel scaffold. Forty-thousand cells/well were seeded atop uncoated TCPS surfaces of a 24-well plate, which formed 2D monolayer and referred to as “scaffold-free 2D culture”. Forty-thousand cells/well were seeded atop ELP1-PEI coated TCPS surfaces of a 24-well plate, which formed 3D spheroids and referred to as “scaffold-free 3D culture”. Cells were cultured in maintenance medium (DMEM supplemented with 10% FBS) for 3 days. Cells were then given endothelial differentiation medium (EGM-2-MV, LONZA, Walkersville, MD, USA) with or without 20 ng/mL or 50 ng/mLVEGF (Peprotech, Rocky Hill, NJ, USA) for 7 days.

### 2.5. Protein and ELISA Assays

For scaffold-free 2D cultures, cells were removed from the TCPS via trypsin digestion. For scaffold-free 3D cultures, cells were rinsed with PBS to remove spheroids. For 3D hydrogel cultures, cells were isolated by degrading the hydrogel using collagenase solution (Sigma, St. Louis, MO, USA). Aliquots were centrifuged for 2 min at 1000 rpm, resuspended in PBS, and sonicated for 30 s at 10% amplitude using a Branson Digital Sonifier 450 (Danbury, CT, USA). Total protein content was determined using the chromatic BCA total protein assay (Thermo Fisher Scientific, Rockford, IL, USA). Protein was also assayed with ELISA assay specific to von Willebrand factor (vWF) (R&D Systems, Minneapolis, MN, USA). All assays were performed per manufacturers’ protocols in triplicate for each of the three replicates of each sample (n = 9).

### 2.6. Acetylated LDL Uptake Assay and Live/Dead Assay

Acetylated LDL uptake was detected by incubating the cultures for 4 h at 37 °C with 2 mg/mL of Ac-LDL labeled with Dil (Invitrogen, Carlsbad, CA, USA). Live/Dead assay was performed as per the manufacturer’s protocol (Invitrogen, Carlsbad, CA, USA). The cultures were visualized with an IX81 microscope (Olympus, Center Valley, PA, USA) with Slidebook imaging software (Intelligent Imaging, Denver, CO, USA).

### 2.7. Statistical Analysis

All experiments were conducted in triplicate. Results are reported as mean ± 95% confidence intervals and analyzed using ANOVA followed by Tukey HSD or Games—Howell post hoc tests in SPSS version 23 statistical software. Values with *p* ≤ 0.05 were deemed statistically significant.

## 3. Results

**Figure 1:**
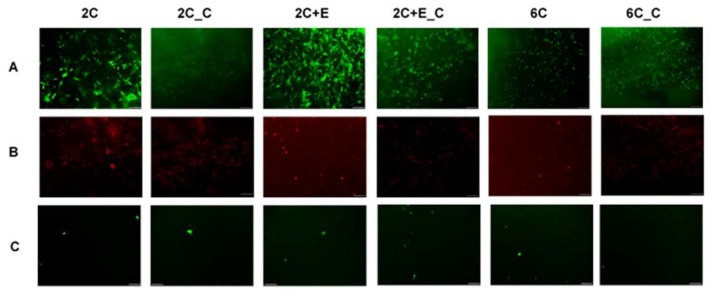
Live/dead assay images depict (row **A**) live and (row **B**) dead cells in various hydrogel conditions on day 7. Uptake of acetylated LDL (green) by cells cultured in various hydrogels on day 7 is shown in row **C**. Scale bars = 100 µm. 2C: 2 mg/mL collagen; 2C_C: the 2C hydrogel cross-linked with EDC/NHS; 2C+E: 2 mg/mL collagen added with 6 mg/mL ELP2; 2C+E_C: the 2C+E hydrogel cross-linked with EDC/NHS; 6C: 6 mg/mL collagen; and 6C_C: the 6C hydrogel cross-linked with EDC/NHS.

**Figure 2:**
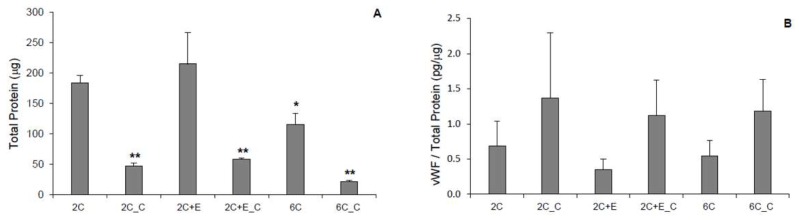
(**A**) Total protein and (**B**) normalized vWF levels (vWF/Total Protein) of cells cultured in various hydrogel conditions on day 7: 2C: 2 mg/mL collagen; 2C_C: the 2C hydrogel cross-linked with EDC/NHS; 2C+E: 2 mg/mL collagen added with 6 mg/mL ELP2; 2C+E_C: the 2C+E hydrogel cross-linked with EDC/NHS; 6C: 6 mg/mL collagen; and 6C_C: the 6C hydrogel cross-linked with EDC/NHS. Bars show the average + upper 95% confidence interval. Statistical analysis performed by ANOVA. * indicates *p* ≤ 0.05 and ** indicates *p* ≤ 0.01 against the 2C hydrogel. n ≥ 8.

When the hASCs were cultured in various hydrogel conditions for 7 days, a significant number of live cells can be seen (Figure 1A). Minimal cell death was seen for the non-cross-linked hydrogels, while some cell death was observed in case of cross-linked hydrogels (Figure 1B). Differences in cell morphology were also evident. Cells appeared to be spread in 2C and 2C+E hydrogels while they appeared spheroidal in 2C_C, 2C+E_C, and 6C_C hydrogels. There appeared to be a mixed morphology of spread and spheroidal cells in the 6C hydrogel (Figure 1A). Protein levels of the cells in each hydrogel condition were assessed after 7 days (Figure 2A). While all cultures were seeded with equal number of cells initially, cultures with a spread morphology exhibited higher levels of protein (~100–200 μg) compared to the cultures with a spheroid morphology (~50 μg) indicating greater proliferation during the 7-day culture period (*p* < 0.05). The 6C hydrogel culture with a mixed morphology showed significantly (*p* < 0.05) less protein (115 ± 19 μg) than the 2C and 2C+E cultures with a spread morphology (183 ± 12 μg and 214 ± 51 μg, respectively), but not to the extent of the 2C_C, 2C+E_C, and 6C_C cultures exhibiting only a spheroid morphology (*p* < 0.05). vWF is a glycoprotein produced by endothelial cells and is commonly used as a marker to characterize differentiated endothelial cells. Angiogenic factors such as VEGF have been shown to upregulate VWF expression in endothelial cells cultured *in vitro* [21]. vWF was detected in each hydrogel condition (Figure 2B). While there appeared to be higher average vWF protein content (1.4 ± 0.9 pg/μg total protein) in the hydrogel conditions containing spheroidal cells compared to hydrogel conditions containing spread cells (0.7 ± 0.4 pg/μg total protein), there was no statistically significant difference among the various conditions. An LDL uptake assay was then conducted for cells in each hydrogel condition. Any hASCs that successfully differentiate into endothelial cells will uptake LDL and fluoresce green in this assay. Only a few such endothelial cells were visualized in each hydrogel condition (Figure 1C).

**Figure 3:**
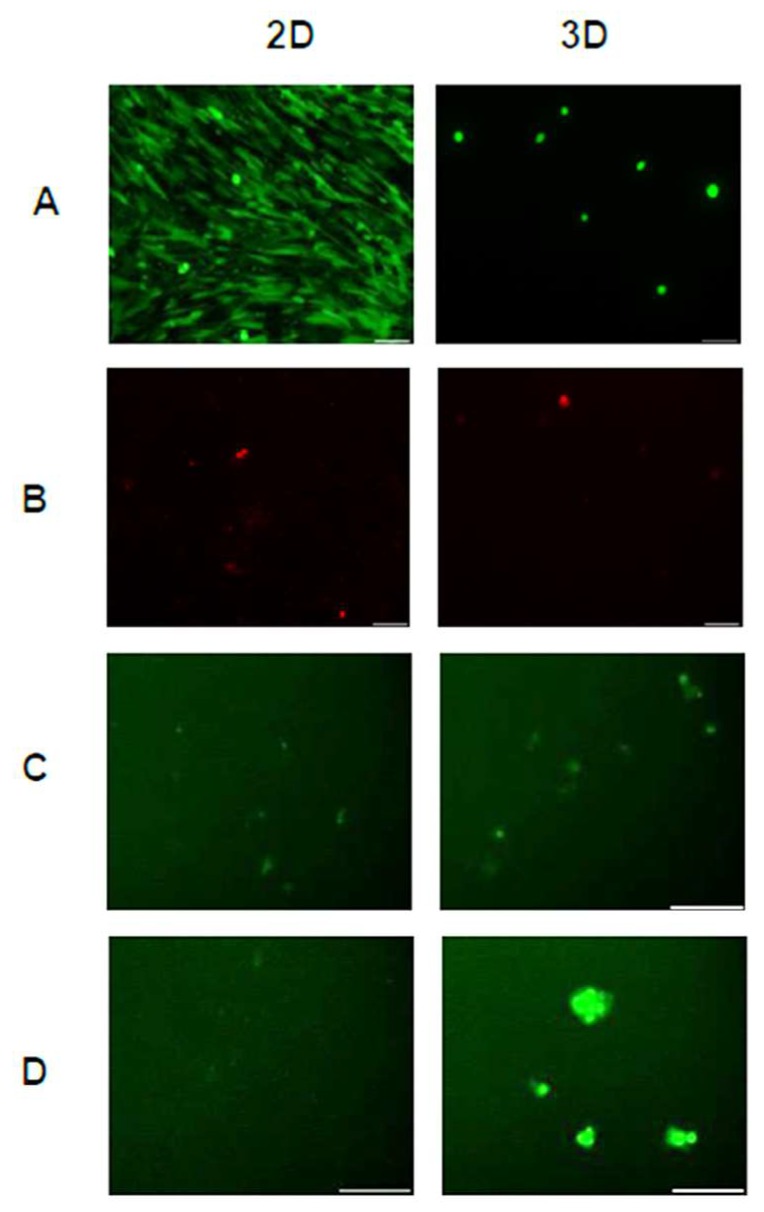
Live/dead assay images depict (**A**) live and (**B**) dead cells in the scaffold-free 2D and 3D conditions on day 7. Uptake of acetylated LDL (green) by cells cultured in 2D and 3D conditions on (**C**) day 0 and (**D**) day 7. Scale bars = 100 µm.

Due to the observed differences in cell morphology (spread versus spheroidal) in the various hydrogel scaffolds, hASCs were cultured on uncoated TCPS surfaces and TCPS surfaces coated with ELP1-PEI. The uncoated TCPS surfaces resulted in a scaffold-free 2D culture with cells having a spread morphology, while the ELP1-PEI coated TCPS surfaces resulted in a scaffold-free 3D culture with cells having a spheroidal morphology (Figure 3A). Cells remained viable throughout the 7-day culture period in both culture conditions, and again, a significant number of live cells can be seen (Figure 3A) with a minimal number of dead cells (Figure 3B).

**Figure 4:**
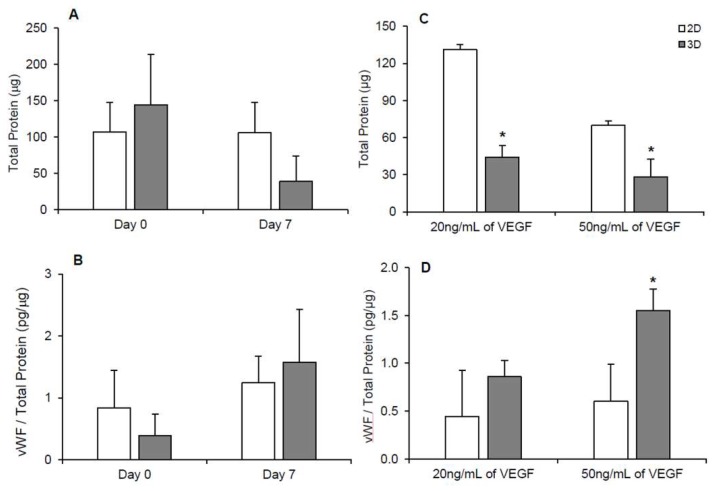
(**A**) Total protein content and (**B**) normalized vWF levels (vWF/total protein) of cells cultured in the scaffold-free 2D and 3D conditions without any additional VEGF supplementation on days 0 and 7. (**C**) Total protein content and (**D**) normalized vWF levels (vWF/total protein) of cells cultured in the scaffold-free 2D and 3D conditions on day 7 with the addition of VEGF. Bars show the average + upper 95% confidence interval. Statistical analysis performed by ANOVA. * indicates *p* ≤ 0.05. n ≥ 7.

Since the live/dead images do not provide a quantitative indication of the cell number, we have quantified the total protein content, which is an indicator of cell number, for the two culture conditions. Protein levels were similar in 2D and 3D scaffold-free conditions on day 0. The protein levels measured (~150 μg) under the scaffold-free conditions (Figure 4A) were statistically similar to those measured in the hydrogel conditions consisting of cells with a spread morphology (Figure 2A). By day 7, the protein levels in the 3D scaffold-free condition (Figure 4A) decreased to levels (~50 μg) consistent with hydrogel conditions with cells of spheroid morphology (Figure 2A). Although this trend was observed, statistically, protein levels remained steady (*p* > 0.05) in 2D and 3D conditions until day 7 (Figure 4A). vWF was also detected in 2D and 3D scaffold-free conditions at days 0 and 7 (Figure 4B) similar to levels (~1 pg/μg total protein) detected in hydrogel conditions (Figure 2B). An apparent increase in vWF, albeit statistically not significant, was observed in 3D scaffold-free condition on day 7 versus the 2D scaffold-free condition (Figure 4B). This increase in vWF was corroborated with a significant presence of endothelial cells visualized utilizing the LDL uptake assay (Figure 3C,D).

**Figure 5:**
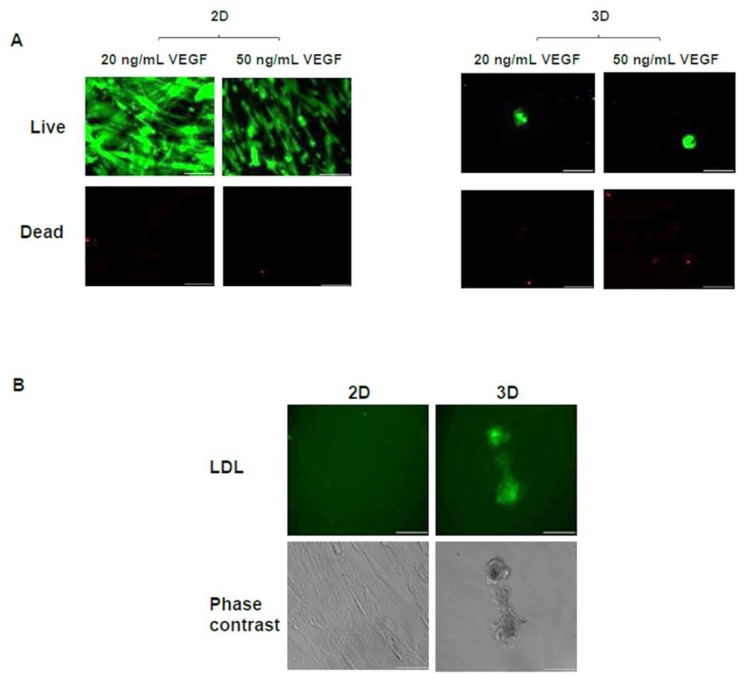
(**A**) Live/dead images of hASCs cultured in the scaffold-free 2D and 3D conditions on day 7. Conditions also indicate the amount of VEGF added to the culture media. Scale bars = 100 µm. (**B**) Uptake of acetylated LDL (green) and phase contrast images in the scaffold-free 2D and 3D conditions given 20 ng/mL of VEGF for 7 days. Scale bars = 100 µm.

To achieve further endothelial differentiation, additional VEGF dosages (20 or 50 ng/mL) were added into the cell culture media of the 2D and 3D scaffold-free conditions. Cells appeared to remain viable throughout the 7 days in 2D and 3D scaffold-free conditions with the addition of VEGF (Figure 5A). With the addition of 20 ng/mL VEGF (Figure 4C), protein levels in the 2D scaffold-free condition (131 ± 9 μg) were significantly (*p* < 0.05) higher than the 3D scaffold-free condition (44 ± 4 μg) on day 7, indicating greater proliferation during the 7-day culture period. Unexpectedly, there was a significant decrease in protein levels of the 2D scaffold free culture from 131 ± 9 μg to 70 ± 14 μg when 50 ng/mL of VEGF was added versus 20 ng/mL of VEGF (*p* < 0.05). On the other hand, protein levels in the 3D scaffold-free condition only slightly reduced to 28 ± 4 μg with the addition of 50 ng/mL VEGF compared to 44 ± 4 μg obtained after the addition of 20 ng/mL VEGF. These protein levels observed after the VEGF addition were similar to those observed without VEGF addition (Figure 4A). The 3D scaffold-free condition showed a similar level of vWF with the addition of 20 ng/mL of VEGF versus the 2D scaffold-free condition (~0.7 pg/μg total protein). The level of vWF in 3D scaffold-free condition (1.5 ± 0.4 pg/μg total protein) was significantly (*p* < 0.05) higher with the addition of 50 ng/mL of VEGF (Figure 4D). Additionally, a larger presence of endothelial cells was visualized by the LDL uptake assay in the 3D scaffold-free condition on day 7 versus the 2D scaffold-free condition with the addition of 20 ng/mL of VEGF (Figure 5B).

## 4. Discussion

It is known that cells, scaffolds, signaling molecules, and blood supply are key factors in regenerative medicine [22]. Unfortunately, clinical applications of tissue engineering are limited by the lack of adequate blood supply [23]. Endothelial cells are a vital component of the newly formed capillaries. There are various stem cell types that can differentiate into endothelial cells. Studies have shown hASCs’ ability to differentiate into various cell types, including endothelial cells, depending on their microenvironment consisting of growth factor cocktail, scaffold formulation, and other culture conditions [24]. Induction of neoangiogenesis-utilizing endothelial cells in hydrogel scaffolds has been a promising tissue engineering strategy. The most commonly used and studied stem cells are embryonic stem cells, somatic or adult stem cells, and induced pluripotent stem cells [2]. However, interest in the potential for hASCs in regenerative medicine is growing due to the plentiful availability of the adipose tissue. Adipose tissue is an abundant, accessible, and replenishable source of adult stem cells that can be isolated from liposuction waste tissue by collagenase digestion and differential centrifugation [25]. Several clinical trials using human adipose stem cell therapy are currently being performed around the world, and there has been a rapid evolution and expansion of their number [26]. Differentiation of hASCs into the endothelial lineage depends on the cell’s microenvironment consisting of the growth factor cocktail, scaffold formulation, and other culture conditions.

In this study, we investigated the ability of hASCs to differentiate into endothelial cells in our 3D spheroid culture systems, namely the collagen and ELP2 containing hydrogels that have shown successful proliferation and differentiation of 3T3-E1 osteoblasts [27] and ELP1-PEI coatings that have shown successful induction of 3D spheroids using 3T3-L1 adipocytes [18] as well as hASCs [19,20]. It should be noted that, ELP1-PEI coating masks the underlying adherent TCPS. The TCPS surfaces without the ELP1-PEI coating form 2D culture, while the TCPS surfaces with the ELP1-PEI coating form the 3D spheroids. While PEI is known to be cytotoxic, we have previously shown that its conjugation with ELP eliminates its cytotoxicity [18]. ELPs are biopolymers that have been widely investigated for biomedical applications. ELPs attempt to recapitulate the extensibility of natural elastin and possess the elastic properties of elastin using a pentapeptide repeat: VPGXG where X is any amino acid besides proline. ELPs have extensibility and thermal properties similar to the natural elastin protein, which is present in blood vessels [10].

Firstly, hASCs were differentiated into endothelial cells in our 3D hydrogel scaffold system. Hydrogel conditions yielded spread and/or spheroidal cells (Figure 1) possibly due to the varying structural/mechanical properties of each hydrogel condition. The cross-linked hydrogels likely possessed a stiffer matrix that prohibited the cells to display spread morphology as well as resulted in reduced proliferative activity. Consequently, protein levels were significantly lower in the cross-linked hydrogel conditions (Figure 2A). Nevertheless, no hydrogel scaffold condition or the spread versus spheroid morphology could be determined as being favorable for hASC differentiation toward the endothelial lineage based on the limited vWF expression (Figure 2B) and LDL uptake (Figure 1C). Therefore, hASCs were exposed to the endothelial differentiation medium in the scaffold-free 2D and 3D spheroid systems atop the uncoated and ELP1-PEI coated TCPS surfaces. Morphological differences were apparent between 2D and 3D scaffold-free conditions. 2D scaffold-free condition was similar to the spread cells seen in the 2C and 2C+E hydrogel scaffolds by exhibiting higher protein levels (Figure 4A). vWF was present in both 2D and 3D scaffold-free conditions (Figure 4B). This implicates that the presence of endothelial differentiation medium was sufficient for cells cultured in 2D scaffold-free condition to possess levels of the mature endothelial marker vWF. However, the 2D scaffold-free condition was not sufficient for cells to uptake LDL (Figure 3).

Interestingly, although the cells in 3D scaffold-free condition were able to uptake LDL (Figure 3), their vWF levels were not statistically different (*p* > 0.05) than the cells in 2D scaffold-free condition (Figure 4B). Since LDL uptake assay indicated a slight increase in hASC differentiation toward the endothelial lineage in our 3D scaffold-free condition, we increased the amount of VEGF in the differentiation medium to determine if further enhancement in the endothelial differentiation could be observed. An increase in vWF levels was seen with the addition of 50 ng/mL and LDL uptake was visualized in 3D scaffold-free condition (Figure 4 and Figure 5). Additionally, branching from the spheroid was observed, that could be explored with future studies to obtain a possible vascular network formation. We posit that increasing the VEGF concentration in the hydrogel scaffold condition will also yield similar results and create an environment for greater endothelial differentiation.

hASCs have been differentiated into endothelial cells in other 3D conditions such as matrigel [28,29], matrigel/fibrin gel [30], and PGLA/PLA mesh [31]. These cells were also able to exhibit tubular microvessels and neovascularization in culture. This is typically detected in culture utilizing phase contrast microscopy. Transplantation of endothelial cells and the 3D matrix into animal models have confirmed that these cells have angiogenic potential to form mature tubular microvessels perfused with blood [30]. Limitations of our study include confirmation of migration, tube formation, and angiogenic potential of our endothelial cells. Nevertheless, adipose-derived stem cells are multipotent cells that can easily be extracted from adipose tissue, are capable of expansion *in vitro*, and have the capacity to differentiate into multiple cell lineages, including endothelial cells, osteoblasts, chondrocytes, myocytes, adipocytes, and neuronal cells. Therefore, our future studies will lead to formation of heterospheroids consisting of multiple cell types which more closely imitate human tissues and even engineering prevascularized constructs with an adequate blood supply to reduce tissue graft failure.

## 5. Conclusions

hASCs were differentiated in endothelial cells in our 3D culture systems. Our data support the hypothesis that hASCs more efficiently differentiate toward the endothelial lineage when formed as spheroids, especially when exposed to higher VEGF concentrations. The increased VEGF concentrations in our culture medium created an environment to mimic conditions in which neoangiogenesis may occur.
